# Morphometric Study of the Mitral and Tricuspid Annuli in Fresh Cadaveric Human Hearts From Uttar Pradesh, India

**DOI:** 10.7759/cureus.117971

**Published:** 2026-10-15

**Authors:** Keerthy L K, Jyoti Chopra, Anurag Rai, Anita Rani, Anoop Verma, Heena Singh

**Affiliations:** 1 Department of Anatomy, King George's Medical University, Lucknow, IND; 2 Department of Thoracic Surgery, King George's Medical University, Lucknow, IND; 3 Department of Forensic Medicine, King George's Medical University, Lucknow, IND

**Keywords:** cadaveric heart, fresh cadaveric hearts, mitral regurgitation, mitral valve annulus, morphometry, north indian population, tricuspid valve annulus, valve reconstruction

## Abstract

Background: Accurate knowledge of mitral and tricuspid annular morphometry is fundamental to the planning of valve repair, annuloplasty, and transcatheter interventions. Population- and ethnicity-specific reference data remain limited for fresh human hearts in Uttar Pradesh.

Objective: This study aims to determine the anteroposterior and transverse diameters of the mitral and tricuspid valve annuli in fresh human cadaveric hearts of the North Indian population and to compare these findings with existing anatomical and echocardiographic literature.

Methods: This cross-sectional analytical study was conducted on 100 fresh human cadaveric hearts. The right atrium and left atrium were opened along standard anatomical planes to expose the tricuspid and mitral annuli, respectively. The anteroposterior and transverse diameters of both annuli were measured using a digital vernier caliper.

Results: The mean anteroposterior and transverse annular diameters of the mitral valve were 2.7 ± 0.4 cm and 2.9 ± 0.3 cm, respectively. The mean anteroposterior and transverse annular diameters of the tricuspid valve were 3.1 ± 0.3 cm and 3.3 ± 0.4 cm, respectively. the mean area of the mitral valve orifice was 6.19 ± 1.34 cm², while that of the tricuspid valve orifice was 8.07 ± 1.70 cm².

Conclusion: This study provides descriptive anatomical reference data for mitral and tricuspid annular dimensions in fresh human cadaveric hearts from the Uttar Pradesh population.

## Introduction

The atrioventricular annulus is the fibrous ring encircling the mitral and tricuspid valve orifices, anchoring the valve leaflets [1]. The mitral annulus lies at the atrioventricular junction and has an elliptical geometry [2]. The tricuspid valve has a D-shaped annulus composed of a flat septal region and curved anterior and posterior regions [3]. Valvular heart disease (VHD) is a significant cause of cardiovascular morbidity and mortality worldwide, of which mitral valve disease is the most common, followed by tricuspid valve disease [4].

Accurate measurement of the mitral and tricuspid annuli is essential for both diagnostic assessment and interventional planning. Deviations from normal annular diameter are indicative of stenosis or regurgitation and congenital heart disease, which can progressively lead to left or right-sided heart failure [5-6]. As such, annular diameter and orifice area serve as key markers of disease severity and help guide treatment decisions. Precise baseline annular dimensions are required for appropriate selection of surgical annuloplasty rings and sizing of transcatheter mitral and tricuspid valve devices [7]. Appropriate valve sizing is essential for device selection and successful transcatheter procedures. Transcatheter valve replacement and annuloplasty devices depend on accurate annular measurements, which are generally CT-derived perimeter and area [8-9].

Few studies have measured valve annular dimensions in fresh, non-formalin-fixed hearts, and fixation can alter tissue dimensions. Our measurements may therefore approximate in vivo anatomy more closely and help fill a gap in the literature. Since in vivo sizing for transcatheter procedures is generally based on CT-derived perimeter and area, these data may also serve as a useful anatomical reference for device sizing. This study aims to measure the mitral and tricuspid annular diameter and orifice area and to highlight their importance in optimizing the outcomes of surgical and transcatheter interventions. It further aims to provide population-specific reference data for the North Indian population.

## Materials and methods

This cross-sectional study was conducted on 100 fresh human cadaveric hearts obtained from autopsy cases at King George's Medical University, Lucknow, Uttar Pradesh, India. Donors of both sexes, aged 20 to 80 years, were included. The study was approved by the Institutional Ethics Committee (Reference No. 3005/Ethics/2024; Reference Code: XXI-PGTSC-IIA/P28). Hearts with an intact tricuspid and mitral valve complex were included. Specimens with gross structural deformities, congenital cardiac anomalies, valve calcification, or infective/endocarditic changes were excluded. All measurements were performed within four hours of death.

An incision was made along the sulcus terminalis to open the right atrium. The atrial wall was then trimmed from posterior to anterior, just above the tricuspid valve annulus. The left atrium was opened and trimmed from anterior to posterior at the level just above the mitral annulus. The mitral and tricuspid valves were then examined from a superior view. Detailed morphometric analysis of the mitral and tricuspid valve annuli was carried out using a digital vernier caliper.

Mitral valve measurements

The anteroposterior (a-b) diameter was measured from the midpoint of the anterior leaflet (a) to the midpoint of the posterior leaflet (b) of the mitral valve. The transverse diameter (c-d) or intercommissural diameter of the MVA was measured from the posteromedial commissure (c) to the anterolateral commissure (d) (Figure 1).

**Figure 1:**
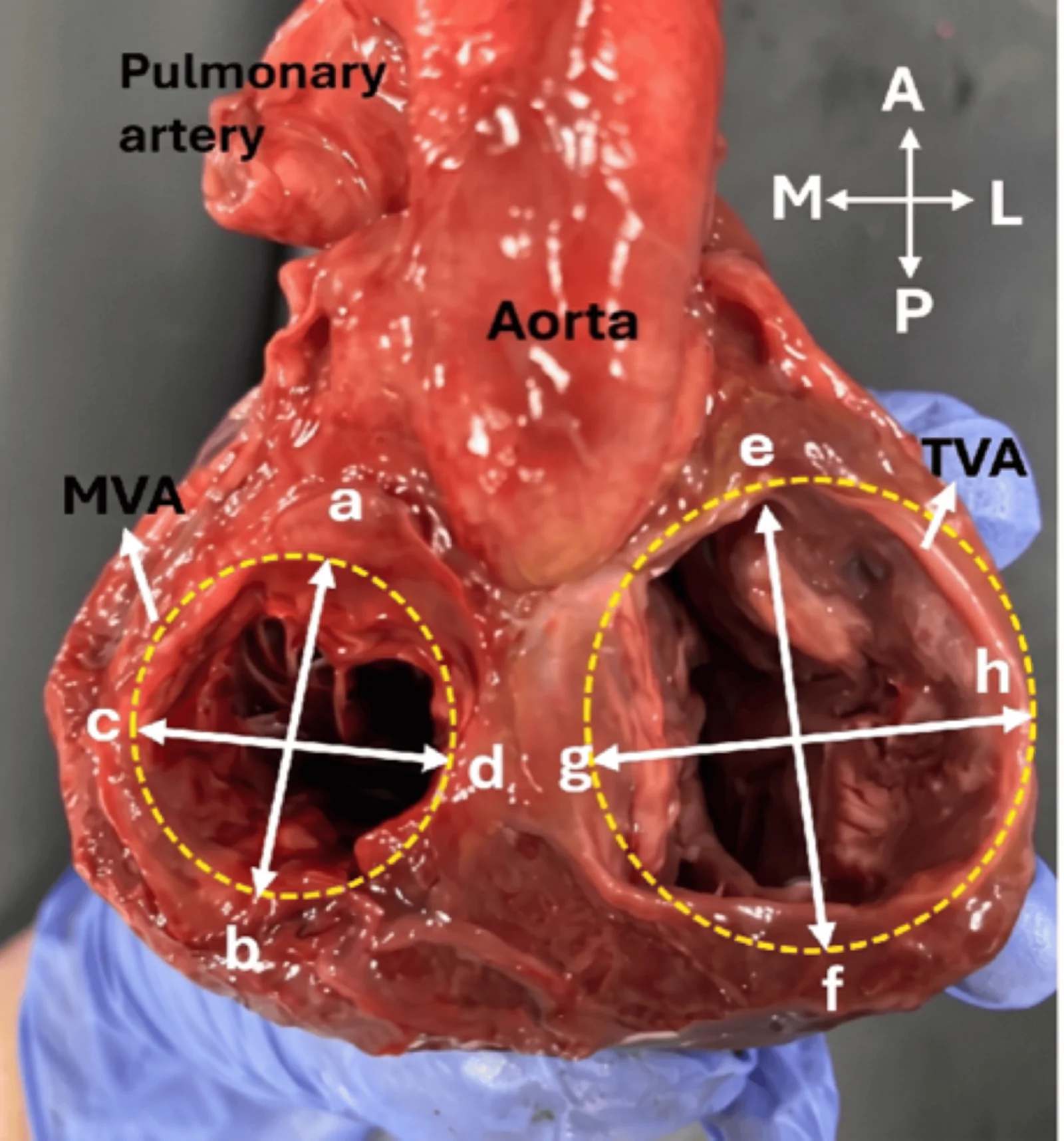
The superior view of heart depicting the methodology used for measuring the anteroposterior and transverse diameter of the MVA and TVA A: anterior; P: posterior; M: medial; L: lateral; MVA: mitral valve annulus; TVA: tricuspid valve annulus (a-b) anteroposterior diameter of the MVA; (c-d) intercommissural diameter of the MVA (c, posteromedial commissure; d, anterolateral commissure); (e-f) anteroposterior diameter of the TVA; (g-h) septolateral diameter of the TVA

Tricuspid valve measurements

The anteroposterior (e-f) diameter was measured from the midpoint of the anterior leaflet (e) to the midpoint of the posterior leaflet (f) of the tricuspid valve. The transverse diameter (g-h) or septolateral diameter was measured from the midpoint of the septal leaflet (g) to the junction of the anterior and posterior leaflets (h) of the tricuspid valve, using a digital vernier caliper (Figure 1).

Statistical analysis

Measurements were recorded in centimeters and expressed as mean ± standard deviation (SD) for each, along with the range, median, and 95% confidence interval (CI). 

## Results

In the present study of 100 human cadaveric hearts, the dimensions of the mitral and tricuspid valve annuli were measured and analyzed. The mean anteroposterior diameter of the mitral valve annulus was 2.7 ± 0.4 cm (median: 2.8 cm; range: 1.7 to 3.6 cm; 95% CI: 2.64 to 2.78 cm), and the mean transverse diameter was 2.9 ± 0.3 cm (median: 3.0 cm; range: 2.0 to 3.6 cm; 95% CI: 2.82 to 2.95 cm). The mean mitral orifice area was 6.19 ± 1.34 cm² (median: 6.45 cm²; range: 2.83 to 9.07 cm²; 95% CI: 5.92 to 6.45 cm²).

The tricuspid valve annulus was larger in all dimensions. Its mean anteroposterior diameter was 3.1 ± 0.3 cm (median: 3.0 cm; range: 2.0 to 4.0 cm; 95% CI: 3.06 to 3.19 cm), and its mean transverse diameter was 3.3 ± 0.4 cm (median: 3.2 cm; range: 2.3 to 4.8 cm; 95% CI: 3.18 to 3.35 cm). The mean tricuspid orifice area was 8.07 ± 1.70 cm² (median: 7.79 cm²; range: 3.61 to 13.51 cm²; 95% CI: 7.74 to 8.41 cm²). These findings are summarized in Table 1.

**Table 1:** Anteroposterior, transverse diameters of the mitral and tricuspid valve annuli and area of the mitral and tricuspid orifice AP: anteroposterior; CI: confidence interval; SD: standard deviation Orifice area calculated per specimen as π/4 × AP × transverse diameter. 95% CI of the mean = mean ± t (0.975, 99) × SD/√n

Parameter	Mean ± SD	Median	Range (min-max)	95% CI of mean
Mitral AP diameter (cm)	2.7 ± 0.4	2.8	1.7-3.6	2.64-2.78
Mitral transverse diameter (cm)	2.9 ± 0.3	3.0	2.0-3.6	2.82-2.95
Mitral orifice area (cm²)	6.19 ± 1.34	6.45	2.83-9.07	5.92-6.45
Tricuspid AP diameter (cm)	3.1 ± 0.3	3.0	2.0-4.0	3.06-3.19
Tricuspid transverse diameter (cm)	3.3 ± 0.4	3.2	2.3-4.8	3.18-3.35
Tricuspid orifice area (cm²)	8.07 ± 1.70	7.79	3.61-13.51	7.74-8.41

## Discussion

The present study evaluated the dimensions of the mitral and tricuspid valves in 100 fresh human cadaveric hearts, contributing to the existing morphometric data on cardiac valve anatomy. Several studies have reported varying measurements for the mitral valve annulus. Khedekar et al. reported an anteroposterior diameter of 27.6 ± 2.2 mm and a transverse diameter of 32.5 ± 2.6 mm in males, compared with 26.2 ± 2.6 mm and 30.6 ± 3.0 mm in females [10]. Our overall mean values fall within this range, and the slightly larger transverse-to-anteroposterior ratio observed in both studies reflects the characteristic elliptical, saddle-shaped geometry of the mitral annulus rather than a true circular configuration, a feature that has consistently been described in prior anatomical and imaging-based literature [10]. Gunnal et al. dissected 116 human cadaveric hearts and reported a mean annular diameter of 2.22 cm and a mean circumference of 9.12 cm, somewhat lower than the present findings [11]. Amgain et al. reported a mean diameter of 2.56 ± 0.32 cm, with the majority of specimens (72%) falling within 2.4-2.7 cm, closely matching the present results [12]. Roy et al. documented a mean annular diameter of 2.6 ± 0.495 cm, further supporting these observations [13]. However, Lama et al. reported a smaller mean diameter (2.01 ± 0.27 cm), possibly pointing to differences in sample size or preservation technique [14]. In an anatomical study of 50 hearts conducted specifically for designing bioprosthesis, Geethanjali et al. reported mitral valve diameters of 3.10 ± 0.40 cm and 3.26 ± 0.48 cm, which align more closely with the present findings than several other cadaveric reports [15]. Echocardiographic studies conducted by Dwivedi et al. demonstrated larger mitral annulus dimensions in vivo, with mean diameters of 3.44 cm in men and 3.11 cm in women at end-systole, likely reflecting dynamic changes during the cardiac cycle [16]. The present cadaveric measurements being static may not capture these physiological variations.

Out of the 100 fresh human hearts in the present study, the mean anteroposterior diameter was 3.1 ± 0.3 cm, and the transverse diameter was 3.3 ± 0.4 cm for the tricuspid valve, which is consistent with some prior studies but differs from others. Premkumar et al. reported a mean tricuspid valve diameter of 26.06 ± 2.49 mm in males and 24.33 ± 1.75 mm in females, somewhat lower than the present measurements [17]. Lama et al. documented a mean diameter of 2.33 ± 0.04 cm, also smaller than the present results [14]. In contrast, Chandouliya et al., in a cadaveric dissection of 100 formalin-fixed hearts, reported a wider range of tricuspid annular diameters (1.8-4.0 cm) with a mean of 3.12 ± 0.47 cm, which is in close approximation with the present results and reinforces the considerable interindividual variability inherent to tricuspid annular anatomy [18]. Dwivedi et al. provided echocardiographic reference values for the tricuspid annulus, reporting 2.84 cm in men and 2.80 cm in women at end-systole, and 3.15 cm in men and 3.01 cm in women at end-diastole, aligning more closely with the present cadaveric findings [16]. This suggests that dynamic in vivo measurements may approximate post-mortem dimensions under certain conditions.

Differences between fresh cadaveric hearts and in vivo imaging (echocardiography) may arise from dynamic cardiac motion. Geographic, ethnic, and gender-based differences may also contribute to anatomical variation. The use of cardiac sizers and imaging-based approaches can further account for variation in reported values.

Limitations 

Measurements taken from static, fresh cadaveric hearts using manual calipers may not fully reflect the dynamic annular dimensions seen on echocardiography during the cardiac cycle. The postmortem tissue changes and the absence of imaging correlation may further limit comparison with in vivo dimensions. The tricuspid annulus has a more complex, nonelliptical, D-shaped configuration, so the calculated area should be interpreted as an approximate geometric estimate rather than an exact anatomical area. In addition, the cadaveric specimens were not stratified by age or sex in the present analysis.

## Conclusions

This study provides descriptive morphometric reference data for the mitral and tricuspid valve annuli in fresh human cadaveric hearts from a North Indian population. The observed measurements contribute to the anatomical understanding of atrioventricular valve annular dimensions and may provide a basis for comparison with imaging-derived measurements. However, their direct application to annuloplasty ring sizing, prosthetic valve selection, or transcatheter intervention planning cannot be established from the present study. Further studies correlating cadaveric measurements with echocardiographic and other cardiac imaging parameters are required to determine their clinical applicability.
